# I’ll Be a Caregiver-Employee: Aging of the Workforce and Family-to-Work Conflicts

**DOI:** 10.3389/fpsyg.2020.00246

**Published:** 2020-02-21

**Authors:** Daniela Converso, Ilaria Sottimano, Sara Viotti, Gloria Guidetti

**Affiliations:** Department of Psychology, University of Turin, Turin, Italy

**Keywords:** caregiver-employees, aging of the workforce, home–work conflict, aging, work sustainability

## Abstract

**Background:**

The double role of *caregiver-employee* (CE) defines those workers who simultaneously serve as an informal, unpaid care provider for sick, disabled, or elderly relatives, and it is a situation that is on the increase in most western countries. Providing informal caregiving can lead to detrimental effects on emotional well-being and several physical and psychological diseases (e.g., caregiver-burden). CEs can suffer double discomfort (at work and at home), but, first of all, they can be exposed to a high level of home-to-work conflict (HWI). In this study, we analyzed the CE phenomenon in a typical Italian public company, where the mean age of workers is particularly high.

**Methods:**

An online questionnaire related to the perception of HWI, well-being, and discomfort at work (depression, emotional exhaustion, job engagement) in relation to the family load (none, parents with <12 children to care for, caregiver to other adults, or children and older adults to care for/old/children to care for employees) was answered by 1704 administrative workers.

**Results:**

More than 20% of our sample was included in the elder caregiver condition or in the double role or “sandwiched” condition with older adults and children to care for. The family load changed significantly between the different age groups: for workers aged between 55 and 64 years, the percentage was nearly 27%. CEs had higher levels of HWI and of personal and job discomfort and lower levels of engagement, when compared with non-CEs. Having “only” older adults to care for (the typical CE condition) was associated with having the most negative results.

**Conclusion:**

This study confirms and underlines the increasing number of CEs in western organizations and their higher levels of HWI, work disengagement, emotional exhaustion, and depression. As the general population and workforce experience increased “graying,” and many more workers become CEs out of necessity, stable caregiver-friendly workplace policies (CFWPs) should be developed.

## Introduction

Aging of the population and, therefore, of the workforce, is related to new conflicts and needs for balance: the traditional work-to-family (WF) and family-to-work (FW) balance and/or conflict (Schilling, 2015; Allen and Martin, 2017) must be reconsidered in the view of different life span challenges and different intergenerational processes within families. Fewer children to raise and more chronically ill relatives (Converso, 2015) and elderly to care for mean that, in Europe, 40% of family caregivers are in paid work (Christensen et al., 2009; Hoffmann and Rodriguez, 2010; Griggs et al., 2019) and fulfill the double role of *caregiver-employee* (CE; Ireson et al., 2018). CE defines those workers who simultaneously serve as an informal, unpaid care provider for sick, disabled, or elderly relatives, and it is a situation that is on the increase in Europe, the United States, Canada, and Japan (Honda et al., 2014; Hilbrecht et al., 2017; Bosco, 2018; Ireson et al., 2018). Because of several factors (i.e., decreased family size, an increase in female employment, reduction of welfare), the spread of CEs is also related to the pension reforms that, in the last decade, have extended working lives until between 67 and 70 years in most western countries. Taking care of elderly (*old*, *old-old*) or chronically ill members of the family is therefore becoming the affair of full-time workers (often *young-old*), rather than the responsibility of retired women/men.

Providing informal caregiving can lead to detrimental effects on emotional well-being and several physical and psychological diseases: caregiver-burden (Moroni et al., 2008; Adelman et al., 2014; Schilling, 2015) is a renowned condition related to long-lasting and/or a high intensity of care that can lead a person to economic loss or social isolation and to higher risks of anxiety and depression, death, and illness than non-caregivers (Schulz and Scott, 1999; Honda et al., 2014). Recently, scholars have reported that informal caregiving can in fact lead to positive impacts on well-being, entailing benefits derived from reciprocity and responsibility, such as satisfaction and meaningfulness (Mortensen et al., 2017; Dich et al., 2019). More generally, it is not the caregiving, but other factors, like the kind of illness and the life stage of the family and of the single caregiver, that can lead to the burden condition (Converso, 2015; Bosco, 2018).

The double condition of CEs has received much attention during the last decade, highlighting a more complex and controversial condition if compared with the condition of unemployed caregivers. In accordance with COR theory (Hobfoll, 1989; Hobfoll et al., 2018), high resource demands – which may occur while providing care – can lead to a loss spiral in the case of additional resources requested in the job domain, while resources gained in one of the two domains may enhance workers’ and/or caregivers’ abilities to cope with the stressors. Moreover, according to role accumulation theory (Sieber, 1974), the double role of paid employee and caregiver can lead to enrichment and buffer the stressors in the other domain. Role strain theory (Goode, 1960), less optimistically, underlines, on the other hand, how double exposure (i.e., a high strain job condition or a high intensity care condition) may easily lead to WH/HW conflict, lower levels of job satisfaction (Rantanen and Kinnunen, 2012; Li et al., 2015; Spagnoli et al., 2019), greater depressive symptoms (Dugan et al., 2016), and negative health consequences (Yeandle et al., 2006). Tement and Korunka (2015) investigated, for example, the impact of types of caregiving on job demands and resources, and the development of WH conflict or enrichment. Mortensen et al. (2017) found that women exposed to high job strain and caregiving had a 34% increased risk of sickness absence compared to women with no high strain and caregiving, but the same effect did not emerge for men. Hansen and Slagsvold (2015) found lower levels of psychological well-being only among women who did not work full-time: in their words, full-time employment could probably offer opportunities to caregivers, while a “double burden” was experienced by those women who combined extensive caregiving and partial employment. Glavin and Peters (2015) found similar results (women/CEs experienced greater health penalties than men/CEs), while in the same country – Canada – no differences between sexes emerged in the study by Hilbrecht et al. (2017): the more time spent caregiving, the lower participants’ well-being ratings were. The mediation of this association by other factors, like time and income adequacy (the more time participants spent caregiving, the lower these resources rated), confirms the complex relationships between roles, resources, and life domains for CEs, and the emerging needs for workplace and social policies and interventions.

### Aims

Despite the rapid growth in the number of CEs in Italy in recent decades, and in light of a culture of family care still strongly linked to female roles, little research on how caregiving is associated with HW interplay has been developed. The aim of the present study was then to pay closer attention to the home-to-work-conflict (HWI) of CEs, focusing particularly on the public sector domain, where the mean age of workers is particularly high and the majority of employees are women: the specificity of this population can therefore highlight today the *scenario* of a wider number of organizations in the near future.

Also, in light of the previously introduced theories (COR; Hobfoll, 1989) that emphasize, on the one hand, the prominent roles of resources and of the anticipation of resource loss as a threat to wellbeing, and, on the other hand [role accumulation and role strain theories; Sieber (1974) and Goode (1960), respectively], the complexity and the non-univocal effects of a double role condition, our research questions were as follows:

(1) What proportions does the CE phenomenon have in a typical Italian public company, and in light of the age and sex of employees?
(2) What is the perception of HWI in relation to the family load and the type of load, and in light of the age and sex of employees?
(3) How do some indicators of well-being and discomfort at work differentiate based on the family load, and in light of the age and sex of employees?

Moreover, with specific welfare policies devoted to CEs in the workplace currently being absent, we considered the basic and common job resources of a relational kind, and explored the buffering effect of social resources at work:

(4) Can social support by superiors and/or colleagues mitigate the discomfort experienced by the CE, and in light of age, sex, and type of family load^1^ ?

Therefore, five hypotheses were tested:

H1: The CE condition is significantly related to age, currently being widely and mostly an adult/eldercare condition.
H2a: The CE condition is accompanied by higher levels of general discomfort (depression) if compared with the non-CE condition.
H2b: The CE condition is accompanied by higher levels of job discomfort (emotional exhaustion) if compared with the non-CE condition.
H2c: The CE condition is accompanied by lower levels of job engagement if compared with the non-CE condition.
H2d: The CE condition is accompanied by higher levels of HWI if compared with the non-CE condition.
H3: HWI is related to the kind of family load, being significantly higher among adult/elder CEs than among employees with children <12.
H4: HWI mediates the relationship between family load and discomfort at work.
H5: Social support moderates the relationship between family load and discomfort at work.

## Materials and Methods

### Participants

A research program was conducted after an agreement between the Unique Guarantee Committee of a large public administration organization in the North of Italy and the Department of Psychology of the University of Turin in 2017. Self-reported questionnaires were administered online at the beginning of 2018. The voluntary nature of participation and the anonymity of the data collection were ensured by the research group of the Department of Psychology. No treatment, including medical, invasive diagnostics, or procedures causing psychological or social discomfort, was administered to the participants. The research also conforms to the 1995 Declaration of Helsinki (as revised in Edinburgh, 2000), and all the contents of the questionnaire were previously approved by the public administration committee that commissioned the project.

The online questionnaire was sent to the 4280 employees of the following administration sectors: Town Council, Commercial Division, Cultural Division, Environment and Civil Protection Division, Technical Service, Mobility Division, Urbanity, Economy Division, Heritage Division, HR, and Decentralization and Services Division. All workers carried out administrative tasks. Finally, 1704 employees (39.81% of the total population of the municipal administrative workers) filled out an online questionnaire on demographic characteristics, family load/caregiving status, HWI, personal discomfort, job discomfort, and job resources. The distribution of gender and age was very similar among respondents and the total population: >60% of municipal workers were women in both groups (60% among the total population and 64% among respondents), and >58% had an age of between 50 and 60 years old (58.6% of the total population and 58.4% of respondents). Moreover, participants were asked about having facilities due to the law 104 (Italian law 104/92), which allow workers who have personal health problems or family members with severe illness or disabilities to care for to abstain from work for 2 h a day or 3 days a month.

### Measures

The measures used were the following:

*HWI*, was measured with the Survey Work–Home Interaction–NijmeGen questionnaire (SWING; Geurts et al., 2005), which contains six items for measuring negative home-to-work interaction (α = 0.83; e.g., “Do you arrive late to work because of domestic obligations?”).

*Depression*, which we considered, in terms of general and personal discomfort, as studies highlight that some natural body changes associated with aging may increase a person’s risk of experiencing depression. To be diagnosed with major depression an individual should present at least four of nine symptoms of depression according to DSM-5. These symptoms are described by the Patient Health Questionnaire 9 (PHQ-9; Kroenke et al., 2001), a renowned tool for diagnosing, monitoring, and determining the severity of depression. The PHQ-9 is suitable for both screening and case-finding: it can be administered by both medical or trained staff and self-administered. It contains nine items (α = 0.88; e.g., “Over the last 2 weeks, how often have you been bothered by any of the following problems? Feeling down, depressed, or hopeless.”).

*Emotional exhaustion* was measured with the Maslach Burnout Inventory (MBI; Schaufeli et al., 1996; Loera et al., 2014; Viotti et al., 2017), which contains five items for emotional exhaustion (α = 0.91; e.g., “I feel emotionally drained by my work.”).

*Engagement* was measured with the Spanish Burnout Inventory (SBI; Gil-Monte and Olivares Faúndez, 2011), which contains five items to measure engagement (α = 0.94; e.g., “I think my job gives me positive experiences.”).

*Social support* was measured with the Job Content Questionnaire (JCQ; Karasek et al., 1998), which contains six items that investigate support from colleagues (α = 0.83; e.g., “People I work with are competent in doing their jobs.”) and four items that investigate support from superiors (α = 0.89; e.g., “My superior pays attention to my work.”).

All test are adapted to the Italian population.

### Statistical Analysis

All statistical analyses were conducted using SPSS 25. Preliminary analyses included descriptive statistics, independent *t*-test, and analysis of variance (ANOVA). Finally, we tested the relationships between family load and depression, emotional exhaustion and engagement, mediated by social support and family to work conflict. The relationships were estimated by moderation-mediation model. The final models included age and gender as control variables. More specifically we used the ANOVA to test the differences between different age group and CE condition of depression, emotional exhaustion, job engagement, and HWI. ANOVA is adopted also to evaluate if HWI is related to the kind of family load.

**FIGURE 1:**
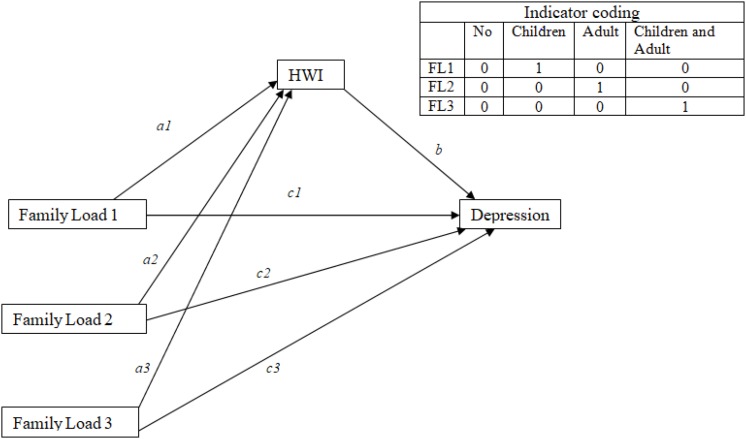
Schematic model of HWI as a mediator between family load and depression (Andrew Hayes’s mediation model, Model 4).

**FIGURE 2:**
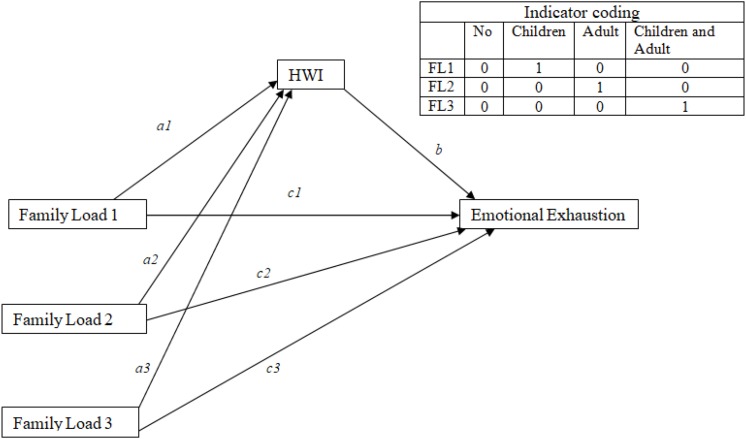
Schematic model of HWI as a mediator between family load and emotional exhaustion (Andrew Hayes’s mediation model, Model 4).

**FIGURE 3:**
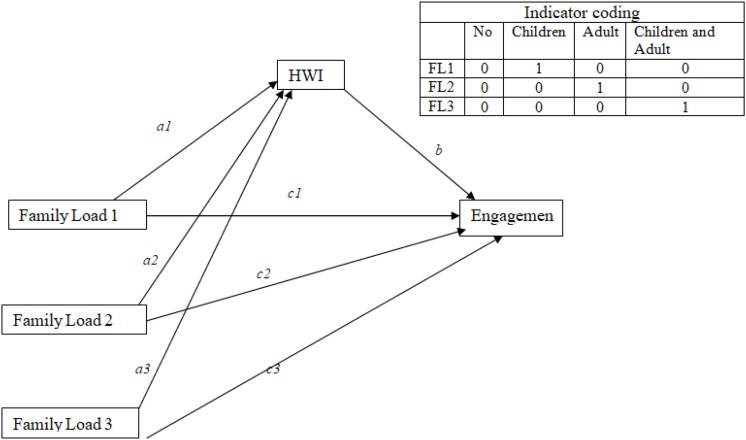
Schematic model of HWI as a mediator between family load and engagement (Andrew Hayes’s mediation model, Model 4).

We tested the mediation role of HWI between the type of family load (the variable family load included four answer option: nobody, children under 12 years of age, elderly relatives, and children and elderly relatives; we considered the independent variable as a multicategorical variable) and depression, exhaustion, and engagement, controlling by age and gender (Figures 1–3). We utilized process by Hayes of SPSS. The model of the mediation analysis was model 4 and refers to situation when the relationship between a predictor variable and an outcome variable can be explained by their relation to a third variable, the mediator variable (Field, 2000). In this case, we consider HWI like a mediator between family load and outcome variables.

Finally, we tested the buffering effect of social resources in the job (support from supervisor and colleagues) on depression, exhaustion, and engagement. Also in this case, we utilized process by Hayes of SPSS. The model of the moderation analysis was model 1.

## Results

The mean age of the working population (administrative workers) was 52.5 years old, and 64.8% of the population were women. Most of the participants were married (64.4%). The CE condition included 11.9% of the participants if we consider those with law facilities for family members’ health problems, whereas 61.7% had no care tasks, 14.8% had children younger than 12, 20.8% had elderly/others to care for, and 2.6% had children and older adults to care for at the same time (Table 1). More specifically, 400 employees had older adults to care for and, among these, 157 (11.9% of the whole sample, 39.3% calculated only on this sub-sample of 400 participants) had also law facilities.

**TABLE 1:** Socio-demographic characteristics.

		*N*	%
Gender	Female	1104	64.8
	Male	600	35.2
Educational level	Primary school	122	7.2
	High school	981	57.9
	Bachelor degree	89	5.3
	Master degree	440	26
	Specialization post master degree	62	3.7
Marital status	Single	301	17.7
	Married	1097	64.4
	Divorced	267	15.7
	Widow	39	2.3
Family load/caregiver status/	No dependent family members	1051	61.7
	Sons and daughters <12 years	253	14.8
	Adults or aged relatives	355	20.8
	Sons and daughters <12 years and adults or aged relatives	45	2.6
Law facilities 104/92	104 for relatives (within the 653 workers with dependent family members to care)	171	26.2
		***M***	***SD***
Age		52.5	7.49

Law facilities – for personal health problems or for family members – were promulgated 30 years ago to mainly face the challenges of severe disabilities and, more sporadically, for eldercare (because of the lower numbers of elderly and the lower age for retirement, since a few years ago, eldercare was mainly managed by households or retired women). This became the main tool for CEs wanting to obtain more flexibility at work. In our sample, having law facilities is directly and significantly associated with age (χ^2^ = 55.02; *p* = 0.002), with the highest number of people with access to them over 51 years old (0.8% among under 40s, the 2.6% among 40–45 years, the 2.9% among 46–50 years, the 11.8% among 51–56 years, and the 10.1% among over 57 years, considering only 653 workers with dependent family members to care).

Table 2 shows that most of the younger workers (<35 years old) have sons or daughters to care for (19.4%), and a few younger workers have dependent adult family members (3.2%). Workers aged between 35 and 44 years old have mainly young children to care for (38.6%); only 6.2% of these workers have dependent elder family members, and 5.1% have both young children and dependent elder family members. As age increases, the situation changes: workers aged between 45 and 54 years old have mainly adult family members to care for (20.4%), and this percentage increases in the age group of those aged between 55 and 64 years old (26.8%). Of workers aged between 45 and 54 years old, 18.6% have younger dependent family members, and this percentage decreases among those aged from 55 to 64 years old (1.3%). The data show that the family load changes in the different age groups (χ^2^ = 428.13; *p* = 0.000).

**TABLE 2:** Frequency distribution of family load per age group.

Age groups											

		<35		35–44		45–54		55–64		>65	
						
		*N*	%	*N*	%	*N*	%	*N*	%	*N*	%
Family load/caregiving status	No dependent family members	23	74.2	111	40.2	312	56.8	588	71.0	17	85.0
	Sons and daughters (<12 years)	6	19.4	134	38.6	102	18.6	11	1.3	0	0.0
	Adults or aged relatives	1	3.2	17	6.2	112	20.4	222	26.8	3	15.0
	Sons and daughters (<12 years) and adults or aged relatives	1	3.2	14	5.1	23	4.2	7	0.8	0	0.0

Considering ANOVA, the analysis show that there is a significant effect of age on HWI and a significant effect of “have dependent family members” on HWI. HWI is always higher among workers with dependent family members and increases with age. Also, depression is higher among workers with dependent family members (with the exception of workers under 40 years) and increases with age. The analysis show that there is a significant effect of age, a significant effect of “have dependent family members” and an interactional effect of age ^∗^ “have dependent family members” on depression. With respect to emotional exhaustion and engagement, only a significant effect of age emerged. Therefore, emotional exhaustion, but also engagement, tend to increase with age, regardless of “have dependent family members.” (Table 3).

**TABLE 3:** Home-to-work conflict (HWI), exhaustion, engagement, and depression by age and dependent family members (mean and ANOVA).

			Descriptive		ANOVA		
				
			*M*	*SD*		*F*	*p*
HWI	<40	No dependent family members	7.65	0.34	Age	4.83	0.001
		Yes dependent family members	8.56	0.33	DFM^1^	30.16	0.000
	40–45	No dependent family members	8.12	0.28	Age * DFM	1.43	0.220
		Yes dependent family members	8.77	0.23			
	46–50	No dependent family members	8.23	0.26			
		Yes dependent family members	8.57	0.25			
	51–56	No dependent family members	8.40	0.13			
		Yes dependent family members	9.45	0.19			
	>57	No dependent family members	8.31	0.12			
		Yes dependent family members	9.59	0.20			
Depression	<40	No dependent family members	4.98	0.65	Age	5.64	0.000
		Yes dependent family members	4.81	0.62	DFM^1^	5.18	0.023
	40–45	No dependent family members	5.15	0.51	Age * DFM	2.51	0.040
		Yes dependent family members	5.29	0.43			
	46–50	No dependent family members	5.02	0.49			
		Yes dependent family members	5.38	0.48			
	51–56	No dependent family members	5.47	0.25			
		Yes dependent family members	7.52	0.35			
	>57	No dependent family members	5.73	0.23			
		Yes dependent family members	6.68	0.38			
Emotional exhaustion	<40	No dependent family members	7.96	1.10	Age	7.24	0.021
		Yes dependent family members	8.36	1.05	DFM^1^	2.16	0.142
	40–45	No dependent family members	8.65	0.88	Age * DFM	1.84	0.119
		Yes dependent family members	9.09	0.74			
	46–50	No dependent family members	9.97	0.84			
		Yes dependent family members	8.76	0.82			
	51–56	No dependent family members	9.78	0.43			
		Yes dependent family members	11.53	0.60			
	>57	No dependent family members	10.47	0.40			
		Yes dependent family members	12.74	0.65			
Engagement	<40	No dependent family members	7.96	1.10	Age	4.18	0.002
		Yes dependent family members	8.36	1.05	DFM^1^	0.76	0.384
	40–45	No dependent family members	8.65	0.88	Age * DFM	2.09	0.080
		Yes dependent family members	9.09	0.74			
	46–50	No dependent family members	9.97	0.84			
		Yes dependent family members	8.76	0.82			
	51–56	No dependent family members	9.78	0.43			
		Yes dependent family members	11.53	0.60			
	>57	No dependent family members	10.47	0.40			
		Yes dependent family members	12.74	0.65			

*^1^DFM is “dependent family members.”*

Considering gender (Table 4), emotional exhaustion is significantly higher among women compared to men (emotional exhaustion: *F* = 40.10; *p* = 0.00) regardless having or not “dependent family members.” Depression is significantly higher among women compared to men (*F* = 33.93; *p* = 0.00) and among workers with dependent family members compared to workers without dependent family members (*F* = 5.38; *p* = 0.00).

**TABLE 4:** Exhaustion and depression for women and men and dependent family members (mean and ANOVA).

		Descriptive			ANOVA	
			
		*M*	*SD*		*F*	*p*
Emotional exhaustion	Female without dependent family members	10.7611	0.32	Gender	40.10	0.00
	Female with dependent family members	11.64	0.40	DFM^1^	1.34	0.25
	Male without dependent family members	8.36	0.42	Gender * DFM	0.75	0.39
	Male with dependent family members	8.49	0.57			
Depression	Female without dependent family members	5.86	0.19	Gender	33.93	0.00
	Female with dependent family members	6.90	0.23	DFM^1^	5.38	0.02
	Male without dependent family members	4.81	0.25	Gender * DFM	2.98	0.08
	Male with dependent family members	4.96	0.33			

*^1^DFM is “dependent family members.”*

Controlling by gender (for exhaustion and depression) and age (for all outcome variables), we tested the influence of the CE conditions and of having facilities provided for by law 104 on psychological health and home–work conflict, among 653 workers with dependent family members. Table 5 highlights that negative home–work interaction (*F* = 4.52; *p* = 0.01), emotional exhaustion (*F* = 5.09; *p* = 0.01), and depression (*F* = 4.52; *p* = 0.01) are higher among workers with adult or older dependent family members. Conversely, engagement does not change significantly in relation to type of family workload but grows among those workers that do not receive facilities by law 104. More in general, CE having 104 facilities present highest HWI, emotional exhaustion, and depression when compared with CE in the same family load condition: law facilities are probably only the indicator of a greater severity of the dependent family members’ conditions, and do not relieve CEs diseases.

**TABLE 5:** Mean of home-to-work conflict, exhaustion, depression, and engagement for type of family load and 104 Italian law facilities controlled by gender (for exhaustion and depression) and age (for all variables) (only considering those workers with dependent family members = 653).

		Descriptive			ANOVA	
				
		*M*	*SD*		*F*	*p*
**Outcome: home-to-work interaction**						
Children dependent family members	With 104	8.37	0.69	FL^1^	5.53	0.00
	Without 104	8.35	0.22	104	8.42	0.00
Adult or older dependent family members	With 104	10.37	0.24	FL * 104	1.85	0.16
	Without 104	9.09	0.21			
Children and older to take care for	With 104	10.77	0.65			
	Without 104	8.77	0.53			
**Outcome: Emotional exhaustion**						
Children dependent family members	With 104	6.93	2.17	FL^1^	5.09	0.01
	Without 104	8.61	0.68	104	0.24	0.62
Adult or older dependent family members	With 104	12.15	0.75	FL * 104	1.01	0.36
	Without 104	11.85	0.66			
Children and older to take care for	With 104	13.31	1.96			
	Without 104	10.21	1.59			
**Outcome: Depression**						
Children dependent family members	With 104	4.54	1.32	FL^1^	4.52	0.01
	Without 104	4.96	0.42	104	1.03	0.31
Adult or older dependent family members	With 104	7.53	0.45	FL * 104	0.64	0.53
	Without 104	6.86	0.40			
Children and older to take care for	With 104	7.82	1.19			
	Without 104	5.94	0.96			
**Outcome: Engagement**						
Children dependent family members	With 104	7.95	1.26	FL^1^	0.00	1.00
	Without 104	10.11	0.40	104	4.29	0.04
Adult or older dependent family members	With 104	8.47	0.43	FL * 104	0.31	0.73
	Without 104	9.58	0.38			
Children and older to take care for	With 104	8.60	1.14			
	Without 104	9.49	0.92			

*^1^Type of family load.*

The results of mediation analyses, where HWI is a mediator between family load and depression (Figure 1), show that the total effect of family load 2 (adult and older to take care) on depression is significant (*B* = 1.64; *p* = 0.00). The significant coefficients of path *a2* (*B* = 1.30; *p* = 0.00) and path *b* (*B* = 0.88; *p* = 0.00) indicate positive associations of family load 2 with HWI and positive associations of HWI with depression. However, the direct effect of family load 2 on depression was not significant. The point estimate of the indirect effect (path *a2* ^∗^ *b*) between family load 2 and depression through HWI is 1.15 (SE = 0.17), and the 95% bias-corrected bootstrap confidence interval is 0.83 to 1.48, which indicates that the indirect effect of family load 2 on depression is statistically significant (Table 6). Data indicated that HWI totally mediated the relationship between family load 2 and depression.

**TABLE 6:** Mediation analyses controlled by age and gender.

Variable	Total effect		Path *c* and *b*		Path *a*		Indirect effect			
				
	*B*	*SE*	*B*	*SE*	*B*	*SE*	*B*	*SE*	LLCI	ULCI
**Outcome variable: Depression**										
Family load 1	−0.42	0.38	−0.62	0.34	0.23	0.21	0.21	0.17	−0.12	0.56
Family load 2	1.64**	0.30	0.49	0.27	1.30**	0.16	1.15	0.17	0.83	1.48
Family load 3	1.28	0.73	0.01	0.64	1.44**	0.40	1.27	0.46	0.44	2.22
HWI	–	–	0.88**	0.04						
*R*^2^	0.05		0.27		0.04					
*F*	15.68		98.08		23.55					
**Outcome variable: Emotional exhaustion**										
Family load 1	−1.07	0.65	−1.36**	0.61	0.26	0.21	0.30	0.23	−0.14	0.75
Family load 2	2.17**	0.51	0.67	0.48	1.32**	0.16	1.50	0.22	1.09	1.98
Family load 3	1.82	1.24	0.17	1.16	1.44**	0.40	1.65	0.58	0.57	2.82
HWI	–	–	1.14**	0.07						
*R*^2^	0.05		0.18		0.05					
*F*	17.29		57.89		16.67					
**Outcome variable: Engagement**										
Family load 1	0.25	0.39	0.34	0.38	0.26	0.21	−0.09	0.08	−0.26	0.04
Family load 2	−0.95**	0.30	−0.45	0.30	1.31**	0.16	−0.50	0.10	−0.71	−0.32
Family load 3	−0.68	0.74	−0.12	0.73	1.44**	0.4	−0.55	0.21	−1.00	−0.18
HWI	–	–	−0.38**	0.04						
*R*^2^	0.01		0.05		0.05					
*F*	3.41		14.76		16.61					

*^∗∗^*p* < 0.01.*

The significant coefficients of path *a3* (*B* = 1.44; *p* = 0.00) indicate also a positive association of family load 3 (adult or older and children to take care) with HWI.

No significance emerges respect family load 1 (children).

The results of mediation analyses, where HWI is mediator between family load and emotional exhaustion (Figure 2), show that the total effect of family load 2 on emotional exhaustion is significant (*B* = 2.17; *p* = 0.00). The significant coefficients of path *a2* (*B* = 1.31; *p* = 0.00) and path *b* (*B* = 1.14; *p* = 0.00) indicate positive associations of family load 2 with HWI and positive associations of HWI with emotional exhaustion. However, the direct effect of family load 2 on emotional exhaustion was not significant. The point estimate of the indirect effect (path *a2* ^∗^ *b*) between family load and emotional exhaustion through HWI was 1.50 (SE = 0.22), and the 95% bias-corrected bootstrap confidence interval was 1.09–1.98, which indicated that the indirect effect of family load on emotional exhaustion was statistically significant (Table 6). Data indicated that HWI totally mediated the relationship between family load 2 and emotional exhaustion.

The significant coefficients of path *a3* (*B* = 1.44; *p* = 0.00) indicate also a positive associations of family load 3 (adult or older and children to take care) with HWI.

About family load 1 (children) emerges only a significant direct effect of family load 1 and emotional exhaustion, which is lower for workers with children (*B* = −1.36; *p* = 0.02).

Finally, the results of mediation analyses, where HWI is mediator between family load and engagement (Figure 3), highlight that the total effect of family load 2 on engagement is significant (*B* = −0.95; *p* = 0.00), and that the significant coefficients of path *a2* (*B* = 1.31; *p* = 0.00) and path *b* (*B* = −0.38; *p* = 0.00) indicate positive associations of family load 2 with HWI and negative associations of HWI with engagement. However, the direct effect of family load 2 on engagement is not significant. The point estimate of the indirect effect (path *a2* ^∗^ *b*) between family load 2 and engagement through HWI was −0.50 (SE = 0.10), and the 95% bias-corrected bootstrap confidence interval is −0.71 to −0.32, which indicates that the indirect effect of family load 2 on engagement is statistically significant (Table 6). Data indicated that HWI totally mediated the relationship between family load 2 and engagement.

The significant coefficients of path *a3* (*B* = 1.44; *p* = 0.00) indicate also positive associations of family load 3 (adult or older and children to take care) with HWI.

No significance emerges respect family load 1.

Table 6 shows that of different family loads, the only one to show a significant impact on the outcome (total effect, net of the mediator) is the one related to elderly to care for, that impacts on depression, exhaustion, and engagement.

The results of the moderation analysis showed that social support from colleagues did not moderate the direct effect of family load (adult/elderly dependent family members) on depression and on emotional exhaustion (Table 7). Also, the social support from superiors did not moderate the direct effect of family load (adult/elderly dependent family members) on depression, on emotional exhaustion, and on engagement (Table 7).

**TABLE 7:** Moderation analyses, controlled by age and gender.

Variable	*B*	*SE*	LLCI	ULCI
**Moderator: Coworkers social support**				

**Output: Depression**				
Family load 1	−0.26	0.37	−0.98	0.47
Family load 2	1.21	0.29	0.64	1.78
Family load 3	0.78	0.73	−0.67	2.22
Social support of colleagues	−1.14	0.14	−1.43	−0.86
Family load 1 * Social support of colleagues	−0.16	0.35	−0.85	0.84
Family load 2 * Social support of colleagues	−0.47	0.28	−1.01	0.07
Family load 3 * Social support of colleagues	−0.67	0.77	−2.18	0.90
**Output: Emotional exhaustion**				
Family load 1	−0.75	0.63	−2.00	0.49
Family load 2	1.59	0.50	0.61	2.57
Family load 3	1.23	1.26	−1.23	3.69
Social support of colleagues	−2.01	0.25	−2.50	−1.53
Family load 1 * Social support of colleagues	−0.61	0.59	−1.78	0.55
Family load 2 * Social support of colleagues	0.06	0.48	−0.87	0.99
Family load 3 * Social support of colleagues	−0.12	1.31	−2.69	2.45

**Moderator: Superiors social support**				

**Output: Depression**				
Family load 1	−0.33	0.38	−1.08	0.41
Family load 2	1.44	0.29	0.86	2.02
Family load 3	0.99	0.73	−0.45	2.44
Social support of colleagues	−1.03	0.14	−1.31	−0.75
Family load 1 * Social support of colleagues	0.04	0.35	−0.65	0.73
Family load 2 * Social support of colleagues	−0.07	0.29	−0.58	0.56
Family load 3 * Social support of colleagues	0.01	0.73	−1.42	1.43
**Output: Emotional exhaustion**				
Family load 1	−0.95	0.64	−2.20	0.31
Family load 2	1.79	0.50	0.80	2.77
Family load 3	0.99	1.25	−1.46	3.43
Social support of colleagues	−1.86	0.24	−2.34	−1.38
Family load 1 * Social support of colleagues	0.21	0.60	−0.96	1.37
Family load 2 * Social support of colleagues	0.02	0.50	−0.99	0.95
Family load 3 * Social support of colleagues	−1.20	1.23	−3.61	1.22
**Output: Engagement**				
Family load 1	0.07	0.36	−0.64	0.78
Family load 2	−0.59	0.28	−1.14	−0.03
Family load 3	−0.13	0.70	−1.51	1.26
Social support of colleagues	1.83	0.14	1.56	2.11
Family load 1 * Social support of colleagues	0.03	0.33	−0.80	0.29
Family load 2 * Social support of colleagues	−0.25	0.28	−1.17	1.57
Family load 3 * Social support of colleagues	0.19	0.69	−2.69	2.45

## Discussion

This study’s aim was threefold. First, we were interested in carrying out a general evaluation of the extent of the CE phenomenon in a large Italian public company, considered as a possible preview of the evolving *scenario* for the public sector (in Italy, as well as in western organizations in general). As expected, in light of the graying of the workforce, >20% of our sample was in the elderly caregiver condition or with a double role, in the “old/children to care for” condition with older adult/children to care for, confirming data similar to those obtained from the U.S. workforce (Tement and Korunka, 2013; Allen and Martin, 2017).

Moreover, family load significantly changed in the different age groups: for workers aged between 55 and 64 years old, the percentage was nearly 27%, while having law facilities was directly and significantly associated with age. Secondly, we investigated the perceptions of HWI, well-being, and discomfort at work in relation to the family load and the type of load. All the considered hypotheses were confirmed: CEs had higher levels of HWI, of general discomfort (depression), of job discomfort (emotional exhaustion), and lower levels of job engagement if compared with non-CEs, confirming, for example, the study of Brannan et al. (2018). Furthermore, HWI [significantly higher among elder CEs than among employed parents and contrary to Tement and Korunka’s (2013), results on WFC] completely mediated the relationship between the type of family load and depression, exhaustion, and engagement in the condition “older adults to care for.”

Interestingly, those having only younger children to care for showed the highest engagement and the lowest depression, emotional exhaustion, and HWI, while the “old/children to care for” group presented higher levels of negative home–work interaction and lower levels of engagement compared to all the others, while depression and emotional exhaustion were significantly higher among the “only” older adults caregiving condition, partly disconfirming those studies that found multiple caregiving responsibilities to lead to more negative consequences than single caregiving ones (Tement and Korunka, 2013). However, this group is very small (only 45 workers); therefore, the data may not highlight particular criticalities due to this limit. Having children appeared then as a protective factor: life is certainly harder to manage when workers have to balance, for example, working hours and school timetables, especially when combined with caring for other adults, but psychological discomfort (emotional exhaustion or depression) is probably buffered by the generative parents’ role and by the forward-thinking perspective that children enhance. Results are then in line with the literature on the topic of generativity (Erikson, 1963; Garcia et al., 2018; that is in some way guaranteed as long as there are children to raise), and consistent with COR theory: children can in fact represent a challenge and be more difficult to care for than the elderly.

Finally, we tested the moderating role of a “classical” relational resource at work, often considered in the WF balance research field: social support from a supervisor and colleagues. Contrary to other studies (Hill, 2005), we did not find significant interaction in any of the considered family load conditions. This result is in line with Tement and Korunka’s (2013) that was focused on work–family conflict and enrichment within the specific condition of elder caregivers: as the authors affirmed, the reason why social support, in particular that from coworkers, is not able to alleviate the demands of caregiving is probably due to the “matching hypothesis” considered by De Jonge and Dormann (2006). Resources might only buffer demands of the same kind, and, in this case, rather than a positive organizational climate and support from coworkers and supervisor, more useful resources would probably be caregiver-friendly policies and services. Moreover, we can assume that sharing the effort of caring for children with colleagues can be supportive and easier to do, while talking about elderly sick people asks for greater reserves or may not be comforting.

### Limitations and Future Research

This study has several limitations, mainly regarding the self-reported measures, the cross-sectional design, and the unique organizational context considered, which does not allow us to generalize results. A longitudinal study could better explain the impact of the CE condition on mental health and on work performance (Zacher et al., 2012). Moreover, future studies should also consider different working populations with different job demands and resources (i.e., different roles for autonomy and skills discretion, or different shifts, workloads, etc.). In this study, other caregiving specificities, like serious disabilities in children, which probably impact on the personal discomfort and work engagement of CEs, were underestimated (see Brannan et al., 2018). Moreover, future research should also consider, for example, the CE’s satisfaction with his/her tasks, which emerged in previous studies as a protective factor for mental health and, therefore, for work performance, for those employees with a high family load (Zacher et al., 2012).

## Conclusion

Despite the limitations, answers to our research questions contribute to CE literature, on the one hand, and to occupational health psychology literature, on the other hand, because of the focus on the interplay between caregiving and the job context; as mentioned elsewhere (Lilly et al., 2012; Adelman et al., 2014), much more attention has been paid to the personal and health side of CEs than to their workers’ condition. Moreover, the research is focused on an “aged” working population, highlighting a still little-known phenomenon, which is the CE condition in a large working population with very similar problems.

This study confirms and underlines the increasing number of CEs in western organizations and their higher levels of HWI, work disengagement, emotional exhaustion, and depression. As literature has demonstrated for a long time (Maslach et al., 1996), low professional accomplishment and high psychological discomfort can lead to absenteeism, early retirements, or reduced work performance (Li et al., 2015; Ireson et al., 2018). A new effect could be the higher complexity of coordinating groups exposed to several limitations because of their family load: as our study revealed, >10% of employees have, for example, law facilities that limit work shifts, mobility, or daily timetables, confirming other studies that highlight, for example, that up to 60% of CEs for Alzheimer older adults are often absent from work for caregiving duties (Bosco, 2018). CEs’ problems may then also impact on employers and collectivity (Schulz and Eden, 2016) and not only on the employees themselves.

An already critical framework can be worsened by the same age of CEs. On the one hand, the lack of support can weaken them, increasing the likelihood of them becoming sick and needing support (Centola, 2016) and vulnerable to stress and depression, with a reduced capability to cope (Li et al., 2015). On the other hand, “aged” CEs can themselves require the need to balance health and work (Gragnano et al., 2017) and have, at the same time, a double load that can reduce their ability to work more quickly, that is going physiologically to be reduced at this life stage (Crawford, 2016) and that, when lowing, involves the increase of disengagement and emotional exhaustion (Converso et al., 2018; Sottimano et al., 2018).

As the general population and workforce experience increasing “graying,” and many more workers become CEs out of necessity, being in paid labor until the later stages of life will require stable caregiver-friendly workplace policies (CFWPs; Ireson et al., 2018), which should therefore be developed and cannot be postponed yet. According to COR theory (Hobfoll, 1989), receiving support in the work domain, when it is harder to receive it inside the family domain when a high load of eldercare is required, could support employees in achieving greater WH balance and, therefore, in sustaining positive energies, mental and physical health, and even work engagement (Russo et al., 2015).

Caregiver-friendly workplace policies can be of the “traditional” kind, like flexible work arrangements, smart or teleworking, and unpaid leave (Zacher and Winter, 2011), improving, for instance, what is already facilitated in Italy with Law 104. Otherwise, support services can be offered (support groups, counseling, workshops, and seminars on caregiving; see Vuksan et al., 2012). Less frequently, but probably more specifically, some employers have introduced adult day care facilities, emergency short-term care, dependent care, and flexible caregiving services (Ireson et al., 2018). This seems to be the most promising prospect.

From an individual point of view, another kind of intervention is related to support generativity (Garcia et al., 2018; Raymund et al., 2018): as we saw before when comparing childcare and eldercare, a higher load regarding children can be a better and proactive challenge, animated, as Erikson (1963) defined generativity, by a concern for guiding the next generation and representing the focal issue (i.e., generativity vs. stagnation) for individuals in the “seventh stage of human development.” Older people cannot represent the same challenge, and more often represent a hindrance demand. As Garcia et al. (2018) highlighted, generativity can then represent a relevant resource for older workers, being related not only to parenthood, but also to a variety of settings, including the workplace. Supporting generativity in the workplace by, for example, assigning mentoring and tutoring roles in the “mature” phase of the working life cycle, could represent, not only an asset for aging workers, but also a resource for those CEs whose family generativity cannot be expressed anymore.

Even if, in our study, social support at work did not emerge as a moderating factor between home–work imbalance and discomfort, it is certainly important to strengthen organizational and supervisor support for reducing the HWI of the caregiver, as highlighted by Li et al. (2015). More than just general and specific support, it could be useful to train supervisors in becoming more supportive of families (Allen and Martin, 2017), even from an *age management* perspective, which should be as specific and attentive as possible to the CE’s condition.

Finally, even unspecific interventions can, in some way, be of certain support for CEs. As Kossek et al. (2017) highlighted, the effects of job interventions on improving psychological health and reducing stress vary according to different non-working caregiving demands: the higher the employee’s need for recovery is (i.e., being in the eldercare or “old/children to care for” care condition compared to the non-eldercare condition), the higher the benefit related to organizational interventions will be.

In conclusion, this study provides empirical evidence for the emerging phenomenon of CEs in an Italian public sector organization that may anticipate a common and widespread condition, suggesting the urgent need for adequate policies.

## Data Availability Statement

The raw data supporting the conclusions of this article will be made available by the authors, without undue reservation, to any qualified researcher.

## Ethics Statement

Ethical review and approval was not required for the study on human participants in accordance with the local legislation and institutional requirements. The patients/participants provided their written informed consent to participate in this study.

## Author Contributions

DC contributed to the conception and design of the study. IS organized the database. IS and SV performed the statistical analysis. DC and IS wrote the first draft of the manuscript. DC, IS, GG, and SV wrote the sections of the manuscript. All authors contributed to the manuscript revision, and read and approved the submitted version of the manuscript.

## Conflict of Interest

The authors declare that the research was conducted in the absence of any commercial or financial relationships that could be construed as a potential conflict of interest.

## Footnotes

1 Family load/caregiving status: The CE condition has been defined by two conditions. The first one, as previously described, asking participants about having facilities due to the Italian law 104/92. As law facilities can be obtained only when a severe health condition is diagnosed, caregiving status was evaluated also asking participants if, in their family, relatives depended on the respondents for their health and care, including children under 12 years old and/or elders or other persons with chronic or severe acute illnesses (the CE condition). Four conditions were therefore considered: employees without a caregiving load (no children younger than 12 and no eldercare); employed parents with children up to 12 years old; employed elder/chronically ill caregivers; and those with a double load of children younger than 12 years old and elders to care for (the “sandwiched” group).
